# Sterile Osteitis and Suppurative Arthritis Associated with Pannus Responding to Colchicine

**DOI:** 10.1155/2013/249471

**Published:** 2013-07-24

**Authors:** Mehmet Engin Tezcan, Özgür Ekinci, Murat Uçar, Berna Göker

**Affiliations:** ^1^Division of Rheumatology, Department of Internal Medicine, Gazi University Medical Faculty, Beşevler, 06500 Ankara, Turkey; ^2^Department of Pathology, Gazi University Medical Faculty, Beşevler, 06500 Ankara, Turkey; ^3^Department of Radiology, Gazi University Medical Faculty, Beşevler, 06500 Ankara, Turkey

## Abstract

Sterile suppurative arthritis is characterized by neutrophilic infiltration of joints without any causative pathogen. Here, we present a 32-year-old man with refractory osteitis and erosive suppurative oligoarthritis with pannus. Treatments with multiple disease modifying antirheumatic drugs were all unsuccessful. However, he had clinical response to colchicine and the synovial hypertrophy and the pannus in the MRI of his left shoulder resolved. In this case, the effects of colchicine on neutrophils might have played a role in treating neutrophilic sterile suppurative arthritis, which, in adults, might be a distinct oligoarticular disease.

## 1. Introduction

Sterile suppurative arthritis is a rare disease, characterized by neutrophilic infiltration of joints similar to septic arthritis, but without any causative pathogen in microbiologic studies [1]. It is also among the descriptive symptoms of pyogenic arthritis, pyoderma gangrenosum, and acne syndrome (PAPA). Sterile suppurative arthritis in PAPA syndrome has been shown to benefit from IL-1 blockade, as well as anti-TNF treatment [2]. Here, we present a patient with osteitis and suppurative erosive arthritis who had a dramatic response to colchicine therapy. 

## 2. Case Presentation

**Figure 1:**
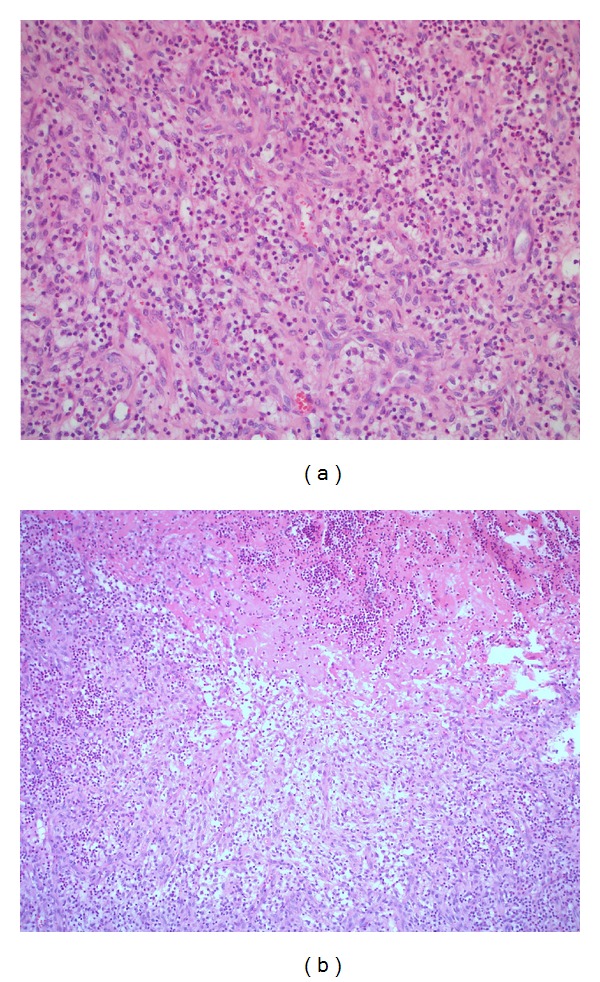
Synovial biopsy. Active inflammation and granulation tissue, predominantly composed of neutrophil leukocytes. Hematoxylin and eosin, ×100 (a) and ×200 (b) magnification.

**Figure 2:**
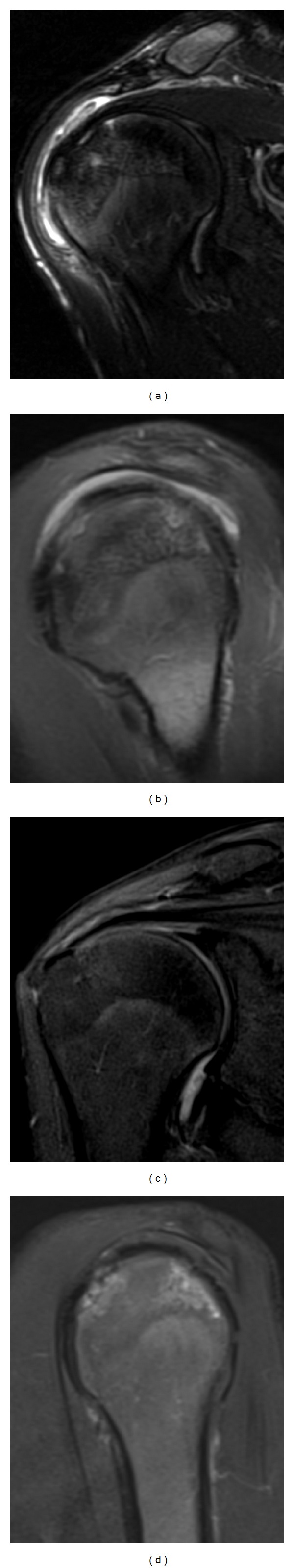
Coronal fat-suppressed T2 weighted (a) and sagittal fat-suppressed proton density weighted (b) sequences demonstrate synovial effusion and pannus in the glenohumeral joint and subdeltoid bursa. Cortical erosions of humeral head are revealed. In the follow-up MR imaging after treatment, coronal fat-suppressed T2 weighted (c) and sagittal fat-suppressed proton density weighted (d) sequences, depict resolution of synovial effusion and pannus. Cortical erosions are still present.

A 32-year-old man, with no significant past medical history except acne in facial region, presented with complaints of intermittent pain and swelling in his right wrist for the last six months. Physical examination was unremarkable except for the swelling, warmth, and tenderness in the right wrist and acneiform lesions on his face. Laboratory evaluation revealed elevated erythrocyte sedimentation rate (ESR) (60 mm/hour) and C-reactive protein (CRP) (24 mg/L); however, rheumatoid factor and anticyclic citrullinated peptide, as well as HLA B27, tests, were found negative. There was no evidence of radiographic sacroiliitis on plain radiography. His skin biopsy from acneiform lesions revealed periadnexal, perivascular, and interstitial neutrophil rich inflammatory infiltrate suggesting acne rosacea. Magnetic resonance imaging (MRI) of the right wrist disclosed contrast enhancement in the distal radius suggesting inflammatory or infectious process, that is, osteomyelitis. Broad spectrum antibiotics, that are effective for both Gram-positive and -negative bacteria, including tetracycline were started. There was no improvement despite treatment with various types of antibiotics for six months. Therefore, synovial biopsy from the right wrist was done. Pathology revealed suppurative arthritis but disclosed no infectious pathogen, such as mycobacteria, fungus, or bacteria (Figure 1). Joint tissue cultures for bacteria, mycobacteria species, and fungus were negative. Bone scintigraphy revealed increased activity in metaphysodiaphysis in his right wrist but it was not considered consistent with infectious osteomyelitis. Three months after the initiation of wrist pain, he began to complain from pain and restriction of motion in his left shoulder. MRI of his left shoulder disclosed synovitis and pannus eroding the head of his humerus (Figures 2(a) and 2(b)). Prednisolone 40 mg/day, hydroxychloroquine 400 mg/day, methotrexate 15 mg/week, and sulfasalazine 2 g/day were started and continued for eighteen months. He had no response to this therapy; therefore, they were discontinued and colchicine 1 mg/day was started. In three months, he had complete resolution of pain and swelling in his wrist and shoulder. His ESR and CRP returned to normal range. His repeat MRI revealed resolution of the synovial hypertrophy and pannus (Figures 2(c) and 2(d)). Due to the impressive response to colchicine therapy, MEFV gene mutations were searched for possibly associated familial Mediterranean fever, but it was negative for the twelve major mutations. 

## 3. Discussion 

We presented a refractory osteitis and erosive suppurative arthritis associated with pannus that was successfully treated with colchicine. Sterile suppurative arthritis is usually a component of PAPA syndrome [2]. However, its association with pannus is not usual. Synovitis, acne, pustulosis, hyperostosis, and sterile osteitis (SAPHO), chronic recurrent multifocal osteomyelitis and PAPA syndrome have osteitis, osteomyelitis, and arthritis, respectively [2–4]. Among these three diseases, only SAPHO syndrome is seen more commonly in adults [3]. However, pannus is not a usual finding in any of these three diseases. Similarly, colchicine is not considered as a major therapeutic agent in any of these three diseases. IL-2 blockers and anti-TNF in PAPA [2], intra-articular steroids, nonsteroidal anti-inflamatory drugs and methotrexate in SAPHO [5], and IL-1 antagonist therapy in chronic recurrent multifocal osteomyelitis have been suggested [6]. Our patient shares several findings with these diseases, such as sterile suppurative inflammation and skin lesions, but pannus in his left shoulder and dramatic response to colchicine are distinctive. 

Colchicine is widely used in rheumatology practice for gout, familial Mediterranean fever, Behçet's disease, pseudogout, and recurring pericarditis with effusion [7]. Its mode of action includes modulation of chemokine and prostanoid production and inhibition of neutrophil and endothelial cell adhesion molecules by which it interferes with the initiation and amplification of the joint inflammation [8]. Colchicine has partial efficacy in SAPHO syndrome [5]. A child with chronic recurrent multifocal osteomyelitis and positive MEFV mutation was successfully treated with colchicine [9]. Our case is MEFV mutation negative. Since the main pathological finding of suppurative arthritis is neutrophilic infiltration, the pannus in our case might be neutrophilic in nature, and the effect of colchicine on neutrophils might have played a crucial role in his dramatic improvement. 

PAPA syndrome has been shown to be associated with a mutation on chromosome 15 that affects proline serine threonine phosphatase-interacting protein (PSTPIP1) or CD2-binding protein 1 (CD2BP1), a tyrosine-phosphorylated protein involved in cytoskeletal organization. This protein has been shown to interact with pyrin, the familial Mediterranean fever (FMF) protein. CD2BP1/PSTPIPI1 is also part of an inflammatory pathway involved in several autoinflammatory syndromes, including FMF [10]. These findings suggest that PAPA syndrome might share some inflammatory pathway with FMF and might explain the impressive response to colchicine. 

In conclusion, neutrophilic sterile suppurative arthritis, osteitis, and skin lesions might be a distinct oligoarticular disease associated with pannus in adults and demonstrate a dramatic response to colchicine therapy.
